# The pathogenesis of COVID-19-induced IgA nephropathy and IgA vasculitis: A systematic review

**DOI:** 10.1016/j.jtumed.2021.08.012

**Published:** 2021-09-28

**Authors:** Hareem Farooq, Muhammad Aemaz Ur Rehman, Abyaz Asmar, Salman Asif, Aliza Mushtaq, Muhammad Ahmad Qureshi

**Affiliations:** Department of Medicine, Mayo Hospital, King Edward Medical University, Lahore, Pakistan

**Keywords:** كوفيد-١٩, اعتلال الكلية بالأيجي أ, التهاب الأوعية بالأيجي أ, فرط نشاط المناعة, الانقلاب المصلي, COVID-19, IgA Nephropathy, IgA Vasculitis, Immune hyperactivation, Seroconversion

## Abstract

**Objective:**

IgA nephropathy (IgAN) and IgA vasculitis (IgAV) are part of a similar clinical spectrum. Both clinical conditions occur with the coronavirus disease 2019 (COVID-19). This review aims to recognize the novel association of IgAN and IgAV with COVID-19 and describe its underlying pathogenesis.

**Methods:**

We conducted a systematic literature search and data extraction from PubMed, Cochrane, ScienceDirect, and Google Scholar following the Preferred Reporting Items for Systematic Reviews and Meta-Analyses (PRISMA) guidelines.

**Results:**

Our search identified 13 cases reporting IgAV and IgAN associated with COVID-19 infection and 4 cases of IgAN following COVID-19 vaccination. The mean, mode, and median ages of patients were 23.8, 4, and 8 years, respectively. Most cases associated with COVID-19 infection were reported in males (77%). Rash and purpura (85%) were the most common clinical features, followed by gastrointestinal symptoms (62%). In symptomatic cases, skin or renal biopsy and immunofluorescence confirmed the diagnosis of IgAN or IgAV. Most patients were treated with steroids and reported recovery or improvement; however, death was reported in two patients.

**Conclusion:**

There is a paucity of scientific evidence on the pathogenesis of the association of IgAN and IgAV with COVID-19, which thus needs further study. Current research suggests the role of IgA-mediated immune response, evidenced by early seroconversion to IgA in COVID-19 patients and the role of IgA in immune hyperactivation as the predominant mediator of the disease process. Clinicians, especially nephrologists and paediatricians, need to recognize this association, as this disease is usually self-limited and can lead to complete recovery if prompt diagnosis and treatment are provided.

## الملخص

**أهداف البحث:**

اعتلال الكلية بالجلوبيولين المناعي "الأيجي أ" والتهاب الأوعية بالجلوبيولين المناعي الأيجي أ لهما طيف سريري متماثل. يتم وصف كلتا الحالتين السريريتين بشكل متزايد مع مرض فيروس كورونا ٢٠١٩ (كوفيد-١٩). تهدف هذه المراجعة إلى التعرف على الارتباط الجديد بين اعتلال الكلية بالأيجي أ والتهاب الأوعية بالأيجي أ وكوفيد-١٩ ووصف أسبابه المرضية الكامنة.

**طرق البحث:**

أجرينا بحثا منهجيا في الأدبيات واستخراج البيانات من الميدلاين، وكوكران، وساينس دايركت، وباحث قوقل العلمي باتباع إرشادات عناصر التقارير المفضلة للمراجعات المنهجية والتحليلات الوصفية (بريزما).

**النتائج:**

حدد بحثنا ١٣ حالة أبلغت عن إصابة اعتلال الكلية بالأيجي أ والتهاب الأوعية بالأيجي أ نتيجة عدوى كوفيد-١٩ وأربع حالات من اعتلال الكلية بالأيجي أ بعد التطعيم كوفيد-١٩. وكان المتوسط والمنوال والوسيط لأعمار المرضى ٢٣.٨و٤ و٨ سنوات على التوالي. تم الإبلاغ عن معظم الحالات المرتبطة بعدوى كوفيد-١٩ لدى الذكور (٧٦.٩٪). كانت الطفح الجلدي والفرفرية (٨٤.٦٪) أكثر المظاهر السريرية شيوعا تليها أعراض الجهاز الهضمي (٦١.٥٪). في حالات الأعراض، أكدت خزعة الجلد أو الكلى واختبار التألق المناعي تشخيص اعتلال الكلية بالأيجي أ أو التهاب الأوعية بالأيجي أ. تم علاج معظم المرضى بالستيرويدات وأبلغوا عن الشفاء أو التحسن، ومع ذلك، تم الإبلاغ عن وفاة مريضين.

**الاستنتاجات:**

هناك ندرة في الأدلة العلمية وهناك حاجة إلى مزيد من الدراسات لفهم التسبب في ارتباط اعتلال الكلية بالأيجي أ والتهاب الأوعية بالأيجي أ بـكوفيد-١٩ بشكل أفضل. تشير الأدبيات الحالية إلى دور الاستجابة المناعية بوساطة الأيجي أ، ويتضح من التحول المصلي المبكر إلى الأيجي أ في مرضى كوفيد-١٩ ودور الأيجي أ في فرط النشاط المناعي باعتباره الوسيط المهيمن لعملية المرض. يحتاج الأطباء، وخاصة أطباء الكلى وأطباء الأطفال، إلى التعرف على هذه العلاقة لأن هذا المرض عادة ما يكون محدودا ذاتيا ويمكن أن يؤدي إلى الشفاء التام إذا تم توفير التشخيص والعلاج الفوريين.

## Introduction

With an incidence of 3–16% in healthy individuals, IgA nephropathy (or Berger's disease) is the most common type of glomerulonephritis across the world.^1^, ^2^, ^3^, ^4^, ^5^ It can be seen more frequently in the second and third decades of life, and the name originates from predominant IgA immune complex deposition in the glomerular mesangium on biopsy.^6^ The classic clinical picture is a child or young adult who develops episode(s) of gross or microscopic haematuria resulting from an upper respiratory tract infection.^2^ It may cause acute renal failure characterized by ankle oedema, facial puffiness, and hypertension. The clinical features are more in line with a nephritic type syndrome, while a nephrotic type rarely occurs in IgA nephropathy.^7^ Closely related to IgA nephropathy is another clinical entity called Henoch Schonlein Purpura (HSP), an IgA-mediated systemic small-vessel vasculitis that, in addition to the kidneys, affects the skin (purpura), joints (arthritis), gut (melena, abdominal pain), etc.^8^^,^^9^ The definitive diagnosis of both can only be made on biopsy and the main distinction between the two is the extra-renal involvement seen in HSP.^2^ Many researchers have upheld the view that both diseases are part of the same spectrum and their underlying pathology is almost identical.^10^^,^^11^

In December 2019, a new viral disease known as COVID-19 was identified. As of May 26, 2021, the World Health Organisation has confirmed more than 167 million cases of this infection on its official website. Although the virus is causing many unknown systemic effects in the human body, it has also been identified as an etiological factor or trigger for some well-recognised clinical entities. Among these conditions, IgA nephropathy and IgA vasculitis (or HSP) are being increasingly described in conjunction with COVID-19. Recent studies have highlighted the role of serum IgA in immune hyperactivation and early seroconversion to IgA in COVID-19 patients.^12^^,^^13^ This evidence may serve as the most plausible explanation for the rise in reported cases of these IgA-mediated diseases, but a comprehensive review that explores this link has not yet been published. Not only does this systematic review serve to elucidate this research question, but it also intends to review other possible pathogenic mechanisms at play. A detailed account of underlying pathogenesis can guide treatment, as well as expand the scientific understanding of researchers at large. A compilation of all such cases will alert practising physicians about rare manifestations of SARS-CoV-2 infection and enhance their knowledge regarding the likely clinical presentation. Timely diagnosis and prompt treatment will improve morbidity and mortality, and ultimately enhance patient care. Given the recent origination of this virus and the paucity of literature on the topic under discussion, a systematic review of cases remains the only reliable medical evidence for researchers and physicians. It also lays a foundation for future researchers as they expand our understanding of this novel clinical association.

## Materials and Methods

### Search strategy

A systematic literature search was conducted (May 29, 2021) on the following four databases: PubMed/MEDLINE, Cochrane, ScienceDirect, and Google Scholar. The search string consisted of a combination of keywords and Mesh terms such as: ‘COVID-19’[MeSH], ‘Covid∗’, ‘SARS-CoV-2’, ‘purpura, schoenleinhenoch’[MeSH], ‘glomerulonephritis, iga’[MeSH], ‘IgA vasculitis’, ‘IgA nephropathy’, ‘Berger’ etc. The complete search string used in each database is provided in the Supplementary files. In order to capture all the available literature, no filter in terms of time, study design, language, country of publication, etc. was used.

### Study selection and data extraction

**Figure 1:**
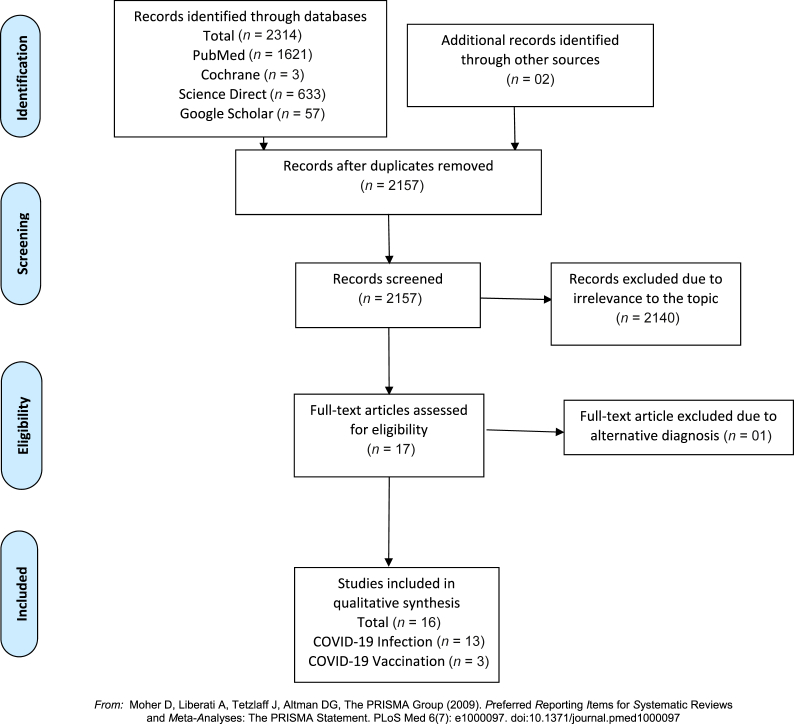
PRISMA flow diagram.

The articles were searched and screened according to the PRISMA flowchart (Figure 1). The records identified through the preliminary search were downloaded into Mendeley and duplicates were removed. Two independent reviewers, HF and MAR, performed the screening and concluded that only case reports and letters to the editor have been published on this topic. In total, 16 articles were shortlisted; 13 articles discussed cases of COVID-19-infection-associated IgAN/IgAV, while another 3 reported COVID-19-vaccine-triggered IgAN. These articles’ bibliographies were sieved to identify any missed cases. All the selected articles were reviewed thoroughly and essential data (e.g. demographics, clinical course, laboratory investigations, and outcome) were extracted and summarised in the form of three tables. Continuous variables are presented as mean, mode, and median, whereas the categorical variables are presented as absolute values and percentages. Microsoft Excel was used for data extraction as well as the calculation of these variables. The references were added through Zotero.

### Quality assessment

The quality of case reports was assessed by Joanna Briggs Institute Critical Appraisal Tool.^14^ Three reviewers (SA, AM, MAQ) first scored each article independently and then awarded a consensus score to each. The score report is provided in the Supplementary files.

## Results

Our search of the four databases identified 2316 articles; 159 were excluded due to duplication and 2140 were removed due to irrelevance to the subject. One article, even though initially considered due to a similar clinical picture to IgAV, was eventually removed as it ruled out IgAV and concluded with a different diagnosis after histological investigations. Finally, 16 articles were selected for inclusion: 13 articles,^15^, ^16^, ^17^, ^18^, ^19^, ^20^, ^21^, ^22^, ^23^, ^24^, ^25^, ^26^, ^27^ including 9 case reports^16^, ^17^, ^18^, ^19^, ^20^, ^21^, ^22^^,^^26^^,^^27^ and 4 letters to the editor,^15^^,^^23^, ^24^, ^25^ reported cases of IgAN and IgAV following COVID-19 infection. The data of these 13 cases are summarized in the form of two tables (Table 1, Table 2), one focusing on notable clinical findings and outcomes, the other on major laboratory investigations. Additionally, three articles^28^, ^29^, ^30^ describing a total of four patients with COVID-19-vaccination-triggered IgAN were found. These are also described in our article to broaden the scope of this review, as the underlying pathogenic mechanisms might be closely linked to COVID-19-infection-related IgAN/IgAV (Table 3).

**Table 1:** Demographics, presentation and outcome of COVID-19 associated IgA Nephropathy and IgA Vasculitis.

Serial No.	Author, Year	Country Reported	Age (years), Gender (M/F)	Notable Medical History	COVID-19 Status	Time between IgAN/IgAV symptoms & COVID-19	Clinical Features (COVID-19)	Clinical Features (Renal)	Clinical Features (Extra-Renal)	Treatment	Outcome	Follow Up
1	Matthieu Allez et al.^15^, 2020	France	24, M	Crohn disease	Ongoing, asymptomatic	Both diagnosed simultaneously	–	–	Skin rash, arthralgia, periarticular swelling, abdominal pain	Steroids, LMWH	Discharged on day 7 on oral steroids & enoxaparin	–
2	Andrea S Suso et al.^16^, 2020	Spain	78, M	Alcohol consumption, HTN, dyslipidemia, aortic stenosis, bladder cancer	Past, resolved	21 days after COVID-19	–	Lower limbs pitting edema, HTN	Wrist arthritis, lower limb purpura	Steroids, rituximab	Serum Cr, urine output & purpura improved but proteinuria & hematuria persisted	–
3	Brett Hoskins et al.^17^, 2021	USA	2, M	None	Ongoing, asymptomatic	Both diagnosed simultaneously	–	–	Abdominal pain, hematochezia, nonbilious emesis with blood streaks, skin rash	Steroids, LMWH	Within 48 h of treatment, cutaneous lesions & abdominal pain improved	1 week after discharge: complete resolution
4	Dalal Anwar AlGhoozi et al.^18^, 2020	Bahrain	4, M	None	Past, resolved	37 days after COVID-19	–	Edema (Ankle)	Pruritic, maculopapular rash, ankle pain	Paracetamol	Discharged the following day, remained pain-free & able to bear weight	1 week after discharge: rash still present, urinalysis normal
5	Nicholas L Li et al.^19^, 2020	Canada	30, M	None	Ongoing, symptomatic	Both diagnosed simultaneously	Fever, runny nose, cough, diarrhea	Frothy urine	Nonbloody diarrhea, abdominal pain, painful purpuric rash, wrist pain	Steroids	Within next 10 days COVID-19 symptoms & rash completely resolved	6 weeks after diagnosis: hematuria & proteinuria on dipstick, RFTs stable, Cr near normal
6	Michal Jacobi et al.^20^, 2021	Israel	3, M	Hirschprung disease	Ongoing, asymptomatic	–	–	–	Mildly dehydration, purpuric rash, abdominal pain, nonbilious emesis	Steroids, empiric antibiotic therapy, IV fluids, NSAIDs	Responded well to steroids & was discharged	–
7	Yi Huang et al.^21^, 2020	China	65, F	HTN, proteinuria, microscopic hematuria & low GFR	Ongoing, symptomatic	7 days before COVID-19	Myalgia, fatigue, headache & cough	Dark colored urine, flank pain, HTN	–	Steroids, valsartan, oseltamivir	Patient became clinically asymptomatic	3 months later: asymptomatic, UACR mildly high, eGFR normal, urine RBC high
8	Simona Gurzu et al.^22^, 2020	United Kingdom	∼1, F	None	Symptomatic, not confirmed on PCR	–	Cough, chills, shortness of breath & fever	–	–	IV fluid boluses, oxygen therapy	Death	Autopsy performed
9	Sunmeet Sandhu et al.^23^, 2020	India	22, M	–	Ongoing, symptomatic	2 days before COVID-19	Fever	Edema	Abdominal pain, vomiting, joint swelling, raised symmetrical lesions on all extremities	Steroids, mycophenolate mofetil	RFTs, LFTs, abdominal & joint symptoms normalised after 2 months	Patient under follow up observation currently
10	Laura Barbetta et al.^24^, 2021	Italy	62, M	–	Ongoing, symptomatic	10 days after COVID-19	Dyspnea, fever	–	Purpuric lesions with raised papules, abdominal pain, vomiting, haematochezia	Bisoprolol, telmisartan, statin, hydroxychloroquine, antibiotics, antivirals, CPAP	Improvement of renal function, progressive remission of abdominal pain & purpura	Referred to outpatient department for follow up
11	Mahdieh Sadat Mousavi et al.^25^, 2020	Iran	6, M	HSP	Symptomatic, not confirmed on PCR	2 days before COVID-19	Fever	Edema	Palpable purpuric maculopapular rash, arthritis, abdominal pain, melena	Steroids, ibuprofen, antibiotics, hydroxychloroquine, cyclophosphamide	Death	–
12	Mayron D. Nakandakari et al.^26^, 2020	Peru	4, F	–	Past, resolved	5 days after COVID-19	Dry cough, rhinorrhea, fever	–	Maculopapular lesions, painful feet, hematemesis, abdominal pain, purpura	Steroids, metamizole, piperazine, antibiotics, ivermectin, omeprazole	Progressive decrease in abdominal pain & purpuric lesions, discharged	–
13	Sarah Falou et al.^27^, 2021	Lebanon	8, M	–	Ongoing, asymptomatic	3 days after COVID-19	–	–	Purpura, ankle pain	IV hydration, NSAIDs, paracetamol	Discharged on 5th day, rash & ankle pain resolved	–

Abbreviations: M Male, F Female, GFR Glomerular Filtration Rate, Cr Creatinine, HSP Henoch Schonlein Purpura, HTN hypertension, RFTs Renal Function Tests, LFTs Liver Function Tests, UACR Urine Albumin to Creatinine Ratio, IV Intravenous, LMWH Low Molecular Weight Heparin, NSAIDs Nonsteroidal Anti-inflammatory Drugs, CPAP Continuous Positive Airway Pressure (−) data not reported.

**Table 2:** Diagnostic and laboratory investigations of COVID-19 associated IgA Nephropathy and IgA Vasculitis.

Serial No	Author, Year	COVID-19 Diagnosis	Relevant Investigations	Renal Function Tests	Urinalysis	Serum IgA levels (g/L)	Renal Biopsy	Renal Electron Microscopy	Skin Biopsy	Immunofluorescence	GI/Abdominal Investigations
1	Matthieu Allez et al.^15^, 2020	RT-PCR	CRP raised D-dimer raised Fibrinogen raised C4 raised	Cr normal	Normal	High (5.3)	–	–	Perivascular & vessel wall infiltration by neutrophils & lymphocytes, leukocytoclasia	Skin biopsy: IgA & C3 positive	CT: circumferential bowel wall thickening & hyperenhancement of the inner mucosa & submucosal edema
2	Andrea S Suso et al.^16^, 2020	IgM/IgG Antibody	Albumin decreased C3 & C4 normal	Cr high	Proteinuria, hematuria with dysmorphic RBCs	Normal	Glomerular sclerosis, segmental mesangial expansion with hypercellularity, epithelial crescents, obliterated glomerular capillary lumens	Electrondense mesangial deposits with podocytes showing extensive pedicular effacement	Cutaneous vasculitis	Renal biopsy: IgA granular deposits	–
3	Brett Hoskins et al.^17^, 2021	RT-PCR	Albumin decreased CRP raised ESR raised	Cr low	Normal	–	–	–	Superficial perivascular inflammation with neutrophils	Skin biopsy: IgA positive	EGD: edema, erythema, superficial erosions in the stomach & duodenum
4	Dalal Anwar AlGhoozi et al.^18^, 2020	RT-PCR	CRP normal ESR normal	Normal	Normal	Normal	–	–	–	–	–
5	Nicholas L Li et al.^19^, 2020	RT-PCR	CRP raised D-dimer raised C3 & C4 normal	Normal	Proteinuria, hematuria	Normal	Focally crescentic & segmentally necrotizing IgAN with focal endocapillary hypercellularity	Mesangial & subendothelial immune-type deposits	Neutrophil rich small-vessel vasculitis	Skin biopsy: IgA, IgG, IgM, C3 negative Renal biopsy: IgA positive	–
6	Michal Jacobi et al.^20^, 2021	RT-PCR	Thrombocytosis Hb decreased Metabolic acidosis	Normal	Normal	–	–	–	–	–	US: increased bowel wall thickness on the left side
7	Yi Huang et al.^21^, 2020	RT-PCR	CRP raised	eGFR low	Proteinuria	High (4.71)	Glomerular sclerosis, fibrocellular crescent, interstitial fibrosis associated with mononuclear inflammation	Mesangial immune deposits	–	Renal biopsy: 2+ granular mesangial staining for IgA, C3, kappa & lambda light chains	
8	Simona Gurzu et al.^22^, 2020	Clinical diagnosis	Hb decreased RBC decreased Hct decreased CRP raised	Urea high Cr low	Leukocytouria	–	Enlarged mesangium with IgA-positive cells, proliferated WT1-positive podocytes, interstitial nephritis with mononuclear cells	–	–	–	
9	Sunmeet Sandhu et al.^23^, 2020	RT-PCR	CRP normal Hb normal	Cr low	Proteinuria	–	Focal necrotizing, mesangial & focal endocapillary proliferative IgAN with mesangial granular deposits of IgA	–	Leukocytoclastic vasculitis	Skin biopsy: IgA positive	US: normal
10	Laura Barbetta et al.^24^, 2021	RT-PCR	–	–	Proteinuria, hematuria, glycosuria, hyaline cast	–	–	–	Perivascular & interstitial lymphocytic infiltrate, extravasated RBCs, ectasic capillaries, endothelial cells with signs of swelling without atypia	Skin biopsy: IgA vascular deposits	CT: enteritis with oedema of the last 40 cm of ileal intestinal tract
11	Mahdieh Sadat Mousavi et al.^25^, 2020	Clinical diagnosis	Hb decreased Leukocytosis CRP raised ESR raised	–	Proteinuria, hematuria	–	–	–	–	–	US: mural thickening of distal ileum, decreased peristalsis.
12	Mayron D. Nakandakari et al.^26^, 2020	IgM/IgG Antibody	Thrombocytosis, aPTT prolonged, Hb, total proteins & albumin decreased	Urea normal Cr low	–	–	–	–	–	–	US: thickened cecum wall with an inflammatory appearance
13	Sarah Falou et al.^27^, 2021	RT-PCR	CRP normal platelets normal	Cr normal	Normal	–	–	–	–	–	–

Abbreviations: RT-PCR Reverse Transcriptase-Polymerase Chain Reaction, CRP C-Reactive Protein, ESR Erythrocyte Sedimentation Rate, Hb Hemoglobin, Hct Hematocrit, Cr Creatinine, RBCs Red Blood Cells, IgAN IgA Nephropathy, US Ultrasound, EGD Esophagogastroduodenoscopy, aPTT Activated Partial Thromboplastin Time, (−) data not reported.

**Table 3:** Demographics, past history, presentation and investigations of COVID-19 vaccine triggered IgA Nephropathy.

Serial No	Author, Year	Country Reported	Age (years), Gender (M/F)	Notable Medical History	Time between 2nd dose & hematuria	Vaccine administered	Clinical Features	Relevant Serum Investigations	Urinalysis	Renal Histology & Immunofluorescence	Comments
1	Hui Zhuan Tan et al.^30^, 2021	Singapore	41, F	Gestational Diabetes	1 day	Pfizer	Hematuria, headache, generalised myalgia	Cr high, IgA high, C3 low	RBCs, protein to creatinine ratio high	Glomeruar IgA staining, focal proliferative glomerulonephritis, mild tubular atrophy & inflammation, mild vessel hyalinosis	Preexisting undiagnosed IgA nephropathy might have been unmasked due to vaccination
2	Lavinia Negrea et al.^29^, 2021	USA	38, F	IgAN	Several hours	Moderna	Body aches, headache, fever, fatigue, chills, gross hematuria	Cr normal	RBCs	–	Exacerbation of preexisting IgAN after vaccination, progressive increase in proteinuria with each dose of vaccine
3	Lavinia Negrea et al.^29^, 2021	USA	38, F	IgAN	Several hours	Moderna	Body aches, headache, fever, fatigue, chills, gross hematuria	Cr normal	RBCs	–	Exacerbation of preexisting IgAN after vaccination, progressive increase in proteinuria with each dose of vaccine
4	Shab E Gul Rahim et al.^28^, 2021	USA	52, F	IgAN	1 day	Pfizer	Gross hematuria, fever, myalgias, body aches, lower back pain	Cr normal	RBCs, protein to creatinine ratio high	–	Exacerbation of preexisting IgAN after 2nd dose of vaccine

Abbreviations: M Male, F Female, Cr Creatinine, IgAN IgA Nephropathy, RBCs Red Blood Cells, USA United States of America (−) data not reported.

For the 13 patients for whom COVID-19-infection-related IgAV/IgAN, was described, the mean age was 23.8 years (range 1–78 years), with the mode and median being 4 and 8 years, respectively. Approximately half the patients belonged to the paediatric population (below 18; *n* = 7, 54%), while six patients were adults (above 18; *n* = 6, 46%). Ten cases were reported in males (77%) and three in females (23%). Ten patients had ongoing COVID-19 infection upon presentation (77%); six patients (46%) were clinically symptomatic whereas four (31%) were asymptomatic with a positive PCR result. In the remaining three patients (23%), COVID-19 infection had resolved before the onset of IgAN/IgAV. This was suggested by either history, previously positive RT-PCR, or now-positive antibody response (IgM/IgG).

The most commonly reported symptoms of IgAV/IgAN were rash/purpura (*n* = 11, 85%), gastrointestinal symptoms, like abdominal pain, melena/haematochezia, haematemesis etc. (*n* = 8, 62%), joint problems/pain (*n* = 7, 54%) and oedema (*n* = 4, 31%). Urinalysis reported proteinuria and haematuria in six (46%) and four (31%) patients, respectively. The cornerstone of definitive diagnosis in all patients was either renal or skin biopsy; abnormal renal biopsy was seen in five cases (39%), whereas skin biopsy abnormalities were reported in six patients (46%). Seven samples (54%) demonstrated positive IgA immunofluorescence: two from kidneys, four from the skin, and one from both the kidneys and the skin.

Immunosuppressants and supportive therapy were the mainstays of treatment. Most (*n* = 9, 69%) patients were treated with steroids, while some patients were also administered antihypertensives, analgesics, and antimicrobials. Among the 12 cases that reported proper outcome/follow-up, 10 (83%) improved significantly with the treatment, whereas death was reported in 2 patients (17%). Both cases of death were reported in the paediatric age group, one in an infant and the other in a child of six years.

Three articles reporting four cases of IgA nephropathy following COVID-19 vaccination have also been described in the literature. All patients were adult females, and the vaccines responsible for this presentation were Moderna and Pfizer (two cases each). Three of these cases occurred as flare-ups in known cases of IgA nephropathy; however, one occurred in a patient who had no previous history of IgAN. The details are summarised in Table 3.

## Discussion

With COVID-19 cases increasing globally, new manifestations of this virus are unfolding before the medical community. This virus of Chinese origin^31^ reportedly affects almost every human organ, thus causing cutaneous, renal, cardiac, psychological, neurological, and even vascular problems.^32^, ^33^, ^34^, ^35^, ^36^, ^37^, ^38^ Though various types of vasculitides and kidney injury have been well reported with COVID-19,^39^^,^^40^ little is known about IgA-mediated systemic vasculitis (Henoch Schonlein Purpura) and nephropathy. With increasing evidence of IgA's role in COVID-19 immune response,^12^^,^^13^ cases of IgA immune complex deposition diseases, like IgA vasculitis and IgA nephropathy, are also rising. There has been a debate among the medical fraternity on the description of IgAV and IgAN as distinct clinical entities, and various specialists consider them part of the same clinical spectrum.^10^^,^^11^

IgA vasculitis characteristically presents with a tetrad of symptoms, including palpable purpura (in absence of concurrent thrombocytopenia or coagulation disorder), arthralgia/joint pain, abdominal discomfort/pain, and renal involvement.^41^ On the other hand, IgA nephropathy is predominantly a renal disease.^42^ The criteria devised by the European League Against Rheumatism (EULAR), Paediatric Rheumatology International Trials Organization (PRINTO), and Paediatric Rheumatology European Society (PRES) are usually employed in the clinical diagnosis of IgAV in children but have limited utility in adult patients. In fact, in order to allow for diagnosis, the presence of purpura along with any of the four features (namely abdominal pain, arthritis, renal disease, or IgA mediated vasculitis/glomerulonephritis) is required.^43^^,^^44^ Although these criteria were not described in all cases per se, the clinical approach used was well in line with them. Rash/purpura was the most common presenting complaint in the cases fulfilling the inclusion criteria of our study, which is consistent with larger clinical studies describing rash as the most common finding in IgAV.^8^^,^^9^ Well in line with the literature,^4^^,^^5^ most cases of IgAN/IgAV associated with COVID-19 were seen in male children or young adults; however, three cases^16^^,^^21^^,^^24^ in old age have been described with SARS-CoV-2, which is rare but also has been reported previously.^1^, ^2^, ^3^, ^4^, ^5^^,^^45^

With regards to pathogenesis, the most widely accepted is the ‘multi-hit hypothesis’. Raised levels of Galactose deficient IgA1 (Gd-IgA1) are crucial for the development of both IgA nephropathy and HSP nephritis. Generation of IgG autoantibodies can be seen targeting these IgA1 immunoglobulins, which leads to the immune complex formation and an inflammatory process; however, the role of the same immune complexes for extrarenal components of HSP is not well established.^46^, ^47^, ^48^, ^49^ For vasculitic/extrarenal components of HSP, a multi-hit model involving IgA1-AECA (anti-endothelial cell antibody) is accepted.^50^ The exact role of COVID-19 in the development of these IgA-related diseases is still being explored, although several possibilities exist. Mucosal infections are believed to enhance IL-6 production that stimulates poor glycosylation/galactosylation of IgA1, thus forming Gd-IgA1 and contributing towards the disease process of IgA vasculitis nephritis (IgAVN) and IgA nephropathy (IgAN).^50^ COVID-19, being a mucosal infection as well, might cause IgAVN and IgAN through this pathway. Studies have revealed that bone marrow is the source of increased IgA1-producing B lymphocytes in patients with IgA nephropathy. The cytokines released in COVID-19 (such as IL-1, IL-6, and TNF) can also potentially lead to the proliferation and maturation of these IgA1-producing B cells, hence leading to IgAN.^51^, ^52^, ^53^

Research is being carried out to document the diagnostic significance of detecting humoral response against SARS-Cov2 infection^54^ and IgA antibodies are emerging as pivotal markers.^55^^,^^56^ Early seropositivity of IgA, emerging two days after initial symptomatology in COVID-19 patients, is being reported in comparison to five days for IgG and IgM.^12^ This might be one of the factors responsible for the formation of immune complexes involving IgA. A previous systematic review exploring the link of COVID-19 with autoimmune diseases has been conducted, suggesting various mechanisms leading to deleterious effects.^57^ The complex genome of this virus and its tendency to mimic molecular machinery enhances its ability to cause autoimmune diseases,^57^^,^^58^ which might be a possible link of this phenomenon with IgAN and IgAV occurring alongside SARS-Cov2 infection.

Moreover, we know that Henoch Schonlein Purpura can be triggered by a variety of other bacterial and viral infections including coxsackievirus, parvovirus, adenovirus, hepatitis A/B, *Staphylococcus aureus*, and group A streptococcus, thus further strengthening our idea of its ominous relationship with coronavirus.^59^, ^60^, ^61^ Evidence also suggests that COVID-19 is capable of inducing endothelial injury as a result of viral components directly affecting endothelial cells via ACE2 receptors, as well as indirectly through inflammation occurring due to defence mechanisms of the host.^62^

COVID-19 infection has also been observed to exacerbate pre-existing IgA nephropathy, as per one of the case reports^21^ included in our study, but the underlying mechanism is debatable. Interestingly, cases of IgA nephropathy also appeared following COVID-19 vaccination in a few individuals.^28^, ^29^, ^30^ Three cases have described flare-ups or worsening of already existing IgA nephropathy following SARS-CoV-2 vaccination, while one case reported appearance of IgAN in a previously healthy patient (although the authors suspected that this patient might have had undiagnosed IgAN). Excessive production of IgA1 monomers in IgAN patients in response to influenza vaccine has been described previously^63^; hence, the possibility that a similar process occurs after COVID-19 vaccination exists. Some scientists are still looking for a plausible explanation regarding the development of IgA nephropathy despite the non-mucosal injection of the vaccine. It has been postulated that in susceptible patients with pre-existing under-galactosylated IgA1 antibodies, the vaccine triggers the production of anti-glycan antibodies that combine with the former and lead to IgAN.^64^

The significance of steroids in treating IgAV and IgAN has been interrogated by various scientists and is said to be controversial.^65^^,^^66^ In our study, most of the patients suffering from COVID-related IgAN and IgAV were subjected to treatment with steroids along with other options available, particularly antibiotics and antihypertensives. As per our results, a favourable outcome was observed in most cases. This is consistent with the understanding that IgAV is a self-limited disease, but it is hard to conclude whether this favourable outcome was due to the self-limiting nature of the disease itself or the efficacy of steroids in treating IgAV.

Based on the evaluation and discussion of the few case reports published so far, the authors would like to emphasize that there are chances of IgAN and IgAV being reported in connection with COVID-19 in the future. Various case reports and reviews have described other forms of vasculitis in COVID-19 too, most commonly Kawasaki disease and some types of leukocytoclastic vasculitis.^67^, ^68^, ^69^ The herculean task of managing this virus is already imposing a burden on healthcare systems worldwide, and associated conditions like IgAV and IgAN can make it all the more challenging. We believe that physicians should take this association into account when examining patients with ongoing or resolved COVID-19 infection who present with symptoms depicting renal pathology, especially patients with a history of hypertension or kidney disease. Timely inspection and treatment would pave the way to improved prognosis of such patients. Furthermore, a focus on more clinical research in this area is needed in order to better understand its incidence and underlying mechanism, as well as providing reliable information in this regard.

The authors would like to acknowledge some limitations as well. We realize that the sample size in our study is small owing to the lack of published articles related to our research question. Since most of the relevant literature includes case reports, it is harder to extrapolate results from the entire population. Serum IgA/Creatinine ratio was not reported in most of the cases, despite its well-known utility in diagnosing IgAN and predicting its outcome.^70^, ^71^, ^72^ The authors independently scored case reports using the Critical Appraisal Tool, so there is a possibility of subjectivity in quality assessment. Lastly, we suspect publication bias, as clinicians are more likely to report clinically significant, unique, and challenging cases.

## Conclusion

IgA-mediated diseases like IgA vasculitis and IgA nephropathy are increasingly occurring in connection with COVID-19. The evidence for the role of IgA in the immune response against COVID-19 is also increasing. The enhancement of IL-6 levels as a result of a mucosal infection like SARS-Cov2 leads to aberrant glycosylation of IgA1 antibodies, forming immune complexes with IgG autoantibodies and depositing in the tissues. Flare-ups/worsening of pre-existing IgAN and new-onset IgAN have also been reported following SARS-CoV-2 vaccination. Special attention must be given by the clinicians to COVID-19 patients belonging to the paediatric age group who present characteristic features of these diseases; however, the possibility of these infections in old age must not be ignored if clinical suspicion exists. Patients may suffer from IgA vasculitis or IgA nephropathy during or even after the resolution of COVID-19 infection, and cases following vaccination have also been reported. Since the vaccination drive and the pandemic are still ongoing, physicians should take common complaints like rash, abdominal pain, and haematuria very seriously. Although most cases are self-limited, timely diagnosis and supportive treatment are still beneficial to prevent long-term consequences to the patient's health.

## Source of funding

This research did not receive any specific grant from funding agencies in the public, commercial, or not-for-profit sectors.

## Conflict of interest

The authors have no conflict of interest to declare.

## Ethical approval

The authors confirm that this review has been prepared in accordance with COPE guidelines and regulations. Given the nature of this article, IRB review was not required.

## Authors' contributions

HF developed the idea of the study, performed a literature search, designed the PRISMA flowchart, analysed the results using Excel, and drafted the manuscript. MAR also developed the idea of the study, performed a literature search, designed the PRISMA flowchart, and drafted the manuscript. AA performed a literature search, interpreted the data after analysing it, and drafted the manuscript. SA generated and filled the tables with data, performed data analysis, and scored case reports on critical appraisal. AM generated and filled the tables with data, interpreted the data, and scored case reports on critical appraisal. MAQ refined the article design, filled the tables with data, interpreted the data, and scored case reports on critical appraisal. All authors have critically reviewed and approved the final draft and are responsible for the content and similarity index of the manuscript.

## References

[1] Galla J.H. (1995 Feb). IgA nephropathy. Kidney Int.

[2] Barratt J., Feehally J. (2005 Jul 1). IgA nephropathy. J Am Soc Nephrol.

[3] Waldherr R., Rambausek M., Duncker W.D., Ritz E. (1989). Frequency of mesangial IgA deposits in a non-selected autopsy series. Nephrol Dial Transplant Off Publ Eur Dial Transpl Assoc - EurRen Assoc.

[4] Li L.-S., Liu Z.-H. (2004 Sep). Epidemiologic data of renal diseases from a single unit in China: analysis based on 13,519 renal biopsies. Kidney Int.

[5] Simon P., Ramee M.-P., Boulahrouz R., Stanescu C., Charasse C., Ang K.S. (2004 Sep). Epidemiologic data of primary glomerular diseases in western France. Kidney Int.

[6] Suzuki K., Honda K., Tanabe K., Toma H., Nihei H., Yamaguchi Y. (2003 Jun). Incidence of latent mesangial IgA deposition in renal allograft donors in Japan. Kidney Int.

[7] Rawla P., Limaiem F. (2021). StatPearls [Internet].

[8] Peru H., Soylemezoglu O., Bakkaloglu S.A., Elmas S., Bozkaya D., Elmaci A.M. (2008 Sep). Henoch Schonlein purpura in childhood: clinical analysis of 254 cases over a 3-year period. Clin Rheumatol.

[9] Trapani S., Micheli A., Grisolia F., Resti M., Chiappini E., Falcini F. (2005 Dec). HenochSchonleinpurpura in childhood: epidemiological and clinical analysis of 150 cases over a 5-year period and review of literature. Semin Arthritis Rheum.

[10] Nicoara O., Twombley K. (2019 Feb 1). Immunoglobulin A nephropathy and immunoglobulin A vasculitis. Pediatr Clin North Am.

[11] Hené R.J., Velthuis P., van de Wiel A., Klepper D., Mees E.J.D., Kater L. (1986 Apr 1). The relevance of IgA deposits in vessel walls of clinically normal skin: a prospective study. Arch Intern Med.

[12] Yu H., Sun B., Fang Z., Zhao J., Liu X., Li Y. (2020 Aug 27). Distinct features of SARS-CoV-2-specific IgA response in COVID-19 patients. Eur Respir J.

[13] Olas K., Butterweck H., Teschner W., Schwarz H.P., Reipert B. (2005 Jun). Immunomodulatory properties of human serum immunoglobulin A: anti-inflammatory and pro-inflammatory activities in human monocytes and peripheral blood mononuclear cells. Clin Exp Immunol.

[14] Moola S., Munn Z., Tufanaru C., Aromataris E., Sears K., Sfetc R., Aromataris E., Munn Z. (2020). JBI manual for evidence Synthesis.

[15] Allez M., Denis B., Bouaziz J.-D., Battistella M., Zagdanski A.-M., Bayart J. (2020 Nov 1). COVID-19–Related IgA vasculitis. Arthritis Rheum.

[16] Suso A.S., Mon C., Alonso I.O., Romo K.G., Juarez R.C., Ramírez C.L. (2020 Nov 1). IgA Vasculitis with nephritis (Henoch−SchönleinPurpura) in a COVID-19 patient. Kidney Int Rep.

[17] Hoskins B., Keeven N., Dang M., Keller E., Nagpal R. (2021 Jan 1). A child with COVID-19 and immunoglobulin A vasculitis. Pediatr Ann.

[18] AlGhoozi D.A., AlKhayyat H.M. (2021 Jan 1). A child with Henoch-Schonlein purpura secondary to a COVID-19 infection. BMJ Case Rep CP.

[19] Li N.L., Papini A.B., Shao T., Girard L. (2021 Jan). Immunoglobulin-A vasculitis with renal involvement in a patient with COVID-19: a case report and review of acute kidney injury related to SARS-CoV-2. Can J Kidney Health Dis.

[20] Jacobi M., Lancrei H.M., Brosh-Nissimov T., Yeshayahu Y. (2021 Feb 1). Purpurona: a novel report of COVID-19-related Henoch-Schonlein Purpura in a child. Pediatr Infect Dis J.

[21] Huang Y., Li X.-J., Li Y.-Q., Dai W., Shao T., Liu W.-Y. (2020 Nov 24). Clinical and pathological findings of SARS-CoV-2 infection and concurrent IgA nephropathy: a case report. BMC Nephrol.

[22] Gurzu S., Satala C.B., Melit L.E., Streinu-Cercel A., Otelea D., Capalna B. (2020). COVID-19 like findings in a fatal case of idiopathic desquamative interstitial pneumonia associated with IgA glomerulonephritis in a 13-month-old child. Front Pediatr.

[23] Sandhu S., Chand S., Bhatnagar A., Dabas R., Bhat S., Kumar H. (2021 Jan). Possible association between IgA vasculitis and COVID-19. Dermatol Ther.

[24] Barbetta L., Filocamo G., Passoni E., Boggio F., Folli C., Monzani V. (2021 Apr). Henoch-Schönleinpurpura with renal and gastrointestinal involvement in course of COVID-19: a case report. Clin Exp Rheumatol.

[25] Mousavi M.S., Jafari M. (2020 Aug 31). COVID-19 in IgA vasculitis. Iran J Pediatr.

[26] Nakandakari Gomez M.D., Marín Macedo H., SeminarioVilca R. (2021 Jan 12). IgA (HenochSchönleinPurpura) vasculitis in a pediatric patient with CO’VID-19 and strongyloidiasis. Rev Fac Med Humana.

[27] Falou S., Kahil G., AbouMerhi B., Dana R., Chokr I. (2021 Mar 29). Henoch Schonlein Purpura as possible sole manifestation of Covid-19 in children. Acta Sci Paediatr.

[28] Rahim S.E.G., Lin J.T., Wang J.C. (2021 Apr 27). A case of gross hematuria and IgA nephropathy flare-up following SARS-CoV-2 vaccination. Kidney Int.

[29] Negrea L., Rovin B.H. (2021 Jun 1). Gross hematuria following vaccination for severe acute respiratory syndrome coronavirus 2 in 2 patients with IgA nephropathy. Kidney Int.

[30] Tan H.Z., Tan R.Y., Choo J.C.J., Lim C.C., Tan C.S., Loh A.H.L. (2021 May 21). Is COVID-19 vaccination unmasking glomerulonephritis?. Kidney Int.

[31] Archived: WHO timeline - COVID-19 [Internet]. Available from: https://www.who.int/news/item/27-04-2020-who-timeline---covid-19;[Accessed 30 May 2021].

[32] Jain U. (2020 Aug 3). Effect of COVID-19 on the organs. Cureus.

[33] Estébanez A., Pérez-Santiago L., Silva E., Guillen-Climent S., García-Vázquez A., Ramón M.D. (2020 Jun). Cutaneous manifestations in COVID-19: a new contribution. J Eur Acad Dermatol Venereol JEADV.

[34] Sachdeva M., Gianotti R., Shah M., Bradanini L., Tosi D., Veraldi S. (2020 May). Cutaneous manifestations of COVID-19: Report of three cases and a review of literature. J Dermatol Sci.

[35] Kanwar D., Baig A., Wasay M. (2020). Neurological manifestations of COVID-19. J Pak Med Assoc.

[36] Serafini G., Parmigiani B., Amerio A., Aguglia A., Sher L., Amore M. (2020 Aug 1). The psychological impact of COVID-19 on the mental health in the general population. QJM Int J Med.

[37] Armaly Z., Kinaneh S., Skorecki K. (2021 Mar 15). Renal manifestations of Covid-19: physiology and pathophysiology. J Clin Med.

[38] Roberts K.A., Colley L., Agbaedeng T.A., Ellison-Hughes G.M., Ross M.D. (2020). Vascular manifestations of COVID-19 - thromboembolism and microvascular dysfunction. Front Cardiovasc Med.

[39] Becker R.C. (2020 Oct 1). COVID-19-associated vasculitis and vasculopathy. J Thromb Thrombolysis.

[40] Dadson P., Tetteh C.D., Rebelos E., Badeau R.M., Moczulski D. (2020). Underlying kidney diseases and complications for COVID-19: a review. Front Med.

[41] Du L., Wang P., Liu C., Li S., Yue S., Yang Y. (2021 Jan). Multisystemic manifestations of IgA vasculitis. Clin Rheumatol.

[42] Tashakkorinia N., Muco E., Tudor M.E. (2021). StatPearls [Internet].

[43] Ozen S., Pistorio A., Iusan S.M., Bakkaloglu A., Herlin T., Brik R. (2010 May 1). EULAR/PRINTO/PRES criteria for Henoch–Schönleinpurpura, childhood polyarteritis nodosa, childhood Wegener granulomatosis and childhood Takayasu arteritis: Ankara 2008. Part II: final classification criteria. Ann Rheum Dis.

[44] Hočevar A., Rotar Z., Jurčić V., Pižem J., Čučnik S., Vizjak A. (2016 Mar 2). IgA vasculitis in adults: the performance of the EULAR/PRINTO/PRES classification criteria in adults. Arthritis Res Ther.

[45] Kaplan B.S., Kaplan B.S., Meyers K.E.C., Bell L.M. (2004). Pediatric nephrology and urology [Internet].

[46] Lau K.K., Suzuki H., Novak J., Wyatt R.J. (2010). Pathogenesis of Henoch-Schönleinpurpura nephritis. Pediatr Nephrol Berl Ger.

[47] Tanaka M., Seki G., Someya T., Nagata M., Fujita T. (2011). Aberrantly glycosylated IgA1 as a factor in the pathogenesis of IgA nephropathy. Clin Dev Immunol.

[48] Wu M.-Y., Chen C.-S., Yiang G.-T., Cheng P.-W., Chen Y.-L., Chiu H.-C. (2018 Aug 20). The emerging role of pathogenesis of IgA nephropathy. J Clin Med.

[49] Suzuki H., Suzuki Y. (2019 Jan 1). Multi-hit pathogenesis of IgA nephropathy. Juntendo Med J.

[50] Heineke M.H., Ballering A.V., Jamin A., Ben Mkaddem S., Monteiro R.C., Van Egmond M. (2017 Dec 1). New insights in the pathogenesis of immunoglobulin A vasculitis (Henoch-Schönleinpurpura). Autoimmun Rev.

[51] Syrjänen J., Hurme M., Lehtimäki T., Mustonen J., Pasternack A. (2002 Mar 1). Polymorphism of the cytokine genes and IgA nephropathy. Kidney Int.

[52] van den Wall Bake A.W.L., Daha M.R., Haaijman J.J., Radl J., van der Ark A., van Es L.A. (1989 Jun). Elevated production of polymeric and monomeric IgA1 by the bone marrow in IgA nephropathy. Kidney Int.

[53] Sugino H., Sawada Y., Nakamura M. (2021 Jul 14). IgA vasculitis: etiology, treatment, biomarkers and epigenetic changes. Int J Mol Sci.

[54] Guo L., Ren L., Yang S., Xiao M., Chang D., Yang F. (2020 Jul 28). Profiling early humoral response to diagnose novel coronavirus disease (COVID-19). Clin Infect Dis.

[55] Sterlin D., Mathian A., Miyara M., Mohr A., Anna F., Claër L. (2021 Jan 20). IgA dominates the early neutralizing antibody response to SARS-CoV-2. Sci Transl Med.

[56] Hasan Ali O., Bomze D., Risch L., Brugger S.D., Paprotny M., Weber M. (2020 Sep 30). Severe coronavirus disease 2019 (COVID-19) is associated with elevated serum immunoglobulin (Ig) A and antiphospholipid IgA antibodies. Clin Infect Dis.

[57] Tang K.-T., Hsu B.-C., Chen D.-Y. (2021). Autoimmune and rheumatic manifestations associated with COVID-19 in adults: an updated systematic review. Front Immunol.

[58] Kim D., Lee J.-Y., Yang J.-S., Kim J.W., Kim V.N., Chang H. (2020 May 14). The architecture of SARS-CoV-2 transcriptome. Cell.

[59] Reamy B.V., Williams P.M., Lindsay T.J. (2009 Oct 1). Henoch-SchönleinPurpura. Am Fam Physician.

[60] Masuda M., Nakanishi K., Yoshizawa N., Iijima K., Yoshikawa N. (2003 Feb). Group A streptococcal antigen in the glomeruli of children with Henoch-Schönlein nephritis. Am J Kidney Dis Off J Natl Kidney Found.

[61] Mandai S., Aoyagi M., Nagahama K., Arai Y., Hirasawa S., Aki S. (2013 Jun 11). Post-Staphylococcal infection Henoch–Schönlein purpura nephritis: a case report and review of the literature. Ren Fail.

[62] Varga Z., Flammer A.J., Steiger P., Haberecker M., Andermatt R., Zinkernagel A.S. (2020). Endothelial cell infection and endotheliitis in COVID-19. Lancet Lond Engl.

[63] van den Wall Bake A.W., Beyer W.E., Evers-Schouten J.H., Hermans J., Daha M.R., Masurel N. (1989 Oct 1). Humoral immune response to influenza vaccination in patients with primary immunoglobulin A nephropathy. An analysis of isotype distribution and size of the influenza-specific antibodies. J Clin Invest.

[64] Abramson M., Yu S.M.-W., Campbell K.N., Chung M., Salem F. (2021 Jul 13). IgA nephropathy after SARS-CoV-2 vaccination. Kidney Med.

[65] Namgoong M., Namgoong M. (2020 Apr 30). Management of IgA vasculitis nephritis (Henoch-Schonleinpurpura nephritis) in children. Child Kidney Dis.

[66] Kurnia B. (2019 Jun 15). Henoch-Schonlein Purpura in children: the role of corticosteroids. Open Access Maced J Med Sci.

[67] Jiao F.-Y. (2020 Jul). Kawasaki disease - a new manifestation of COVID-19 in children. Chin J Contemp Pediatr.

[68] Kumar G., Pillai S., Norwick P., Bukulmez H. (2021 Apr 1). Leucocytoclastic vasculitis secondary to COVID-19 infection in a young child. BMJ Case Rep CP.

[69] Gómez M.C., González-Cruz C., Ferrer B., Barberá M.J. (2020 Oct 1). Leucocytoclastic vasculitis in a patient with COVID-19 with positive SARS-CoV-2 PCR in skin biopsy. BMJ Case Rep CP.

[70] Gong W., Liu M., Luo D., Liu F., Yin L., Li Y. (2019 Dec). High serum IgA/C3 ratio better predicts a diagnosis of IgA nephropathy among primary glomerular nephropathy patients with proteinuria ≤ 1 g/d: an observational cross-sectional study. BMC Nephrol.

[71] Tomino Y., Suzuki S., Imai H., Saito T., Kawamura T., Yorioka N. (2000). Measurement of serum IgA and C3 may predict the diagnosis of patients with IgA nephropathy prior to renal biopsy. J Clin Lab Anal.

[72] Zhang J., Wang C., Tang Y., Peng H., Ye Z.C., Li C.C. (2013 Feb). Serum immunoglobulin A/C3 ratio predicts progression of immunoglobulin A nephropathy. Nephrology.

