# Supplementary material for: The pathogenesis of COVID-19-induced IgA nephropathy and IgA vasculitis: A systematic review
Source: J Taibah Univ Med Sci. 2021 Sep 28;17(1):1–13. doi: 10.1016/j.jtumed.2021.08.012 (PMC8479423; doi:10.1016/j.jtumed.2021.08.012)
Supplement: Multimedia component 1 [file mmc1.docx]

**Search Strategy**

**Concept 1: COVID-19**

**Keywords: “**COVID*” OR “COVID19” OR “covid 19” OR “corona*” OR “SARS-CoV-2”

**MeSH:** "COVID-19"[Mesh]

**Search Strategy:**  "COVID-19"[Mesh] OR **“**COVID*” OR “COVID19” OR “covid 19” OR “corona*” OR “SARS-CoV-2”

**Concept 2: IgA Glomerulonephritis**

**Keywords:** “IgA vasculitis” OR “IgA nephropathy” OR “berger*”

**MeSH:** ("Purpura, Schoenlein-Henoch"[Mesh]) OR "Glomerulonephritis, IGA"[Mesh]

**Search Strategy:** ("Purpura, Schoenlein-Henoch"[Mesh]) OR "Glomerulonephritis, IGA"[Mesh] OR “IgA vasculitis” OR “IgA nephropathy” OR “berger*”

**Database wise search strategy:**

**PubMed Strategy: (1621 articles)**

("COVID-19"[MeSH Terms] OR "covid*"[All Fields] OR "COVID19"[All Fields] OR "COVID-19"[All Fields] OR "corona*"[All Fields] OR "SARS-CoV-2"[All Fields]) AND ("purpura, schoenlein henoch"[MeSH Terms] OR "glomerulonephritis, iga"[MeSH Terms] OR "IgA vasculitis"[All Fields] OR "IgA nephropathy"[All Fields] OR "berger*"[All Fields])

**Google Scholar Search Strategy (57 articles)**

("covid 19") AND (("igA vasculitis") OR ("henoch schonlein purpura"))

**ScienceDirect Search Strategy: (633 articles)**

("COVID 19") AND ("henoch schoenlein purpura" OR "IgA glomerulonephritis" OR "IgA vasculitis" OR "IgA nephropathy" OR "berger")

**Cochrane Search Strategy (3 reviews)**

Search Name: COVID 19 and IgA Nephropathy

Date Run: 29/05/2021 22:27:15

Comment:

ID Search Hits

#1 MeSH descriptor: [COVID-19] explode all trees 337

#2 covid (Word variations have been searched) 5632

#3 coronavirus (Word variations have been searched) 2999

#4 SARS-CoV-2 (Word variations have been searched) 2011

#5 MeSH descriptor: [Purpura, Schoenlein-Henoch] explode all trees 52

#6 MeSH descriptor: [Glomerulonephritis, IGA] explode all trees 247

#7 IgA vasculitis (Word variations have been searched) 25

#8 IgA nephropathy (Word variations have been searched) 613

#9 Berger (Word variations have been searched) 3141

#10 #1 OR #2 OR #3 OR #4 5352

#11 #5 OR #6 OR #7 OR #8 OR #9 3803

#12 #10 AND #11 03
