# Supplementary material for: The pathogenesis of COVID-19-induced IgA nephropathy and IgA vasculitis: A systematic review
Source: J Taibah Univ Med Sci. 2021 Sep 28;17(1):1–13. doi: 10.1016/j.jtumed.2021.08.012 (PMC8479423; doi:10.1016/j.jtumed.2021.08.012)
Supplement: Multimedia component 2 [file mmc2.docx]

**Scoring of Case Reports on Critical Appraisal**

| **Study Author, Year** | **Final Score (/8)** | **Included/Excluded** | **Additional Comments** |
| --- | --- | --- | --- |
| Matthieu Allez et al., 2020 | 6 | Included | Follow up of the patient is not mentioned |
| Andrea S Suso et al., 2020 | 8 | Included | Case thoroughly described |
| Brett Hoskins et al., 2021 | 8 | Included | Skin biopsy is not mentioned but it is accounted for by gastric biopsy |
| Dalal Anwar AlGhoozi et al., 2020 | 8 | Included | Symptoms, Investigation, treatment, follow up mentioned completely. |
| Nicholas L Li et al., 2020 | 7 | Included | Sufficiently satisfactory |
| Michal Jacobi et al., 2021 | 7 | Included | No skin biopsy attempted despite cutaneous symptoms |
| Yi Huang et al,2020 | 7 | Included | Did not describe the type of test that was used to detect COVID-19 |
| Simona Gurzu et al., 2020 | 7 | Included | COVID-19 diagnosis was not confirmed, as PCR was negative and serological test was unavailable. It was only suspected due to clinical presentation |
| Sunmeet Sandhu et al., 2020 | 8 | Included | Blood Urea Nitrogen and blood in urine were not described. IgA levels not mentioned |
| Laura Barbetta et al.,2021 | 7 | Included | Past medical history wasn’t described and serum IgA wasn’t mentioned |
| Mahdieh Sadat Mousavi et al.,2020 | 6 | Included | No biopsy or IgA levels mentioned to support HSP diagnosis, it was only clinical |
| Mayron D. Nakandakari et al.,2020 | 7 | Included | Patient demographics less clear |
| Sarah Falou et al.,2021 | 7 | Included | No skin/renal biopsy or serum IgA was mentioned to diagnose HSP in full spectrum |

**COVID-19 vaccine triggered IgA Nephropathy**

| Study Author, Year | Final Score(/8) | Included/Excluded | Additional Comments |
| --- | --- | --- | --- |
| Hui Zhuan Tan et al, 2021 | 8 | Included | Very detailed and comprehensive |
| Lavinia Negrea et al, 2021 | 7 | Included | There was no mention of the dates at which vaccines were administered |
| Lavinia Negrea et al, 2021 | 7 | Included | There was no mention of the dates at which vaccines were administered |
| Shab E Gul Rahim et al, 2021 | 7 | Included | There was no mention of dates at which vaccination was done. |
